# Analysis of risk factors for revision in distal femoral fractures treated with lateral locking plate: a retrospective study in Chinese patients

**DOI:** 10.1186/s13018-020-01850-z

**Published:** 2020-08-12

**Authors:** Guojin Hou, Fang Zhou, Yun Tian, Hongquan Ji, Zhishan Zhang, Yan Guo, Yang Lv, Zhongwei Yang, Yawen Zhang

**Affiliations:** Department of Orthopaedic Surgery, Peking University Third Hospital, No 49, North Garden Road, HaiDian District, Beijing, 100191 China

**Keywords:** Distal femoral fracture, Periprosthetic fracture, Total knee arthroplasty, Locking plate, Lateral, Revision, Risk factors

## Abstract

**Background:**

To analyze the risk factors of revision operation after the treatment of distal femoral fracture with lateral locking plate (LLP).

**Methods:**

Retrospective analysis of the clinical data of 152 cases with distal femoral fracture treated in our hospital from March 2005 to March 2019. The SPSS 26.0 software (univariate analysis and logistic regression analysis) was used to analyze the general condition, fracture-related factors, operation-related factors, and construct characteristics of internal fixation.

**Results:**

Sixteen of 152 patients who were included in the study underwent revision surgery, with a revision rate 10.5%. Univariate analysis showed that there were significant differences in age, body mass index (BMI), fracture type, supracondylar involved or not, type of incision, quality of reduction, ratio of length of plate/fracture area (R1), the ratio of the length of the plate/fracture area above the condylar (R2), ratio of distance between proximal part of fracture and screw/working length of proximal plate (R3) between the two groups (*P* < 0.05). Logistic regression analysis showed that age [OR for age > 61.5 group is 4.900 (1.071–22.414)], fracture type [OR for A3 fracture is 8.572 (1.606–45.750), the OR for periprosthetic fracture after TKA is 9.073 (1.220–67.506)], poor reduction quality [OR is 7.663 (1.821–32.253)], and the ratio of the length of the plate/fracture area above the condylar were the possible risk factors (*P* < 0.05).

**Conclusion:**

Age, fracture type (A3 and periprosthetic fracture after TKA), poor reduction quality, and the ratio of the length of the plate/fracture area above the condylar were the possible risk factors of the revision in distal femoral fractures treated with lateral locking plate. The appropriate application of the locking plate and operation strategy are the key to reduce the revision rate in distal femoral fractures.

## Background

The incidence of distal femoral fracture accounting for 4–6% of femoral fractures [1]. The distribution of patient’ s age is bimodal, the younger patients are mostly caused by high energy injury, while the older patients are mostly combined with osteoporosis and low energy mechanism such as falls from standing height. For both groups, surgical treatment of distal femoral fracture should fully consider many factors, such as the patient’s physical condition, bone stock, pattern and position of fracture, articular surface involvement, comminution degree, and the presence of an adjacent implant. At present, there are many kinds of internal fixators available, such as 95° angle plate, dynamic condyle plate, lateral locking plate (LLP) of distal femur, and retrograde intramedullary nail. LLP has become increasingly popular since the technique was introduced in the late 1990s for its minimally invasive implantation, less soft tissue/blood supply destruction, and advantages of angle stability [2, 3]. However, with the accumulation of cases, initial success rates of the treatment of distal femoral fractures with LLP have given way to high incidence of complications 32%, such as delayed union, nonunion, and failure of internal fixation, among which the incidence of nonunion could be as high as 0–21% [4, 5]. This increase may be multifactorial and attributable to an increased use of the technique, which is an application to a broader range of patient types. Distal femoral nonunions are disastrous and associated with axial malalignment, chronic pain, loss of ambulatory function, and decreased knee range of motion (ROM) [6].

The purpose of this study was to retrospectively analyze the clinical date of patients admitted to our hospital and to identify patient characteristics, injury, first operation, and construct characteristics that are independent predictors of increased risk of revision when LLP is used to treat distal femoral fractures. Using this data, a model was built to predict which patients admitted with distal femoral fracture would need revision. Measures to promote healing such as medical intervention, early bone grafting, and medial plate addition may be implemented when high-risk cases were identified. By better managing this process, patients may receive optimal medical care without catastrophic complications.

## Materials and methods

### Patient data

This retrospective study was based on data gathered from the hospital electronic medical record (EMR) system and the blood bank database. Approval was taken from the local research committees. Clinical data of the patients with distal femoral fracture treated in our hospital from March 2005 to March 2019 were analyzed. The revision was defined as the need for reoperation due to nonunion or failure of internal fixation.

Inclusion criteria were the following: fresh fracture (< 3 weeks), age > 18 years old, treated with LLP, and follow-up data before fracture healing. Exclusion criteria were as follows: old fracture (> 3 weeks), age < 18 years old, pathological fracture, AO/OTA type 33-B fracture, and no follow-up data.

**Fig. 1:**
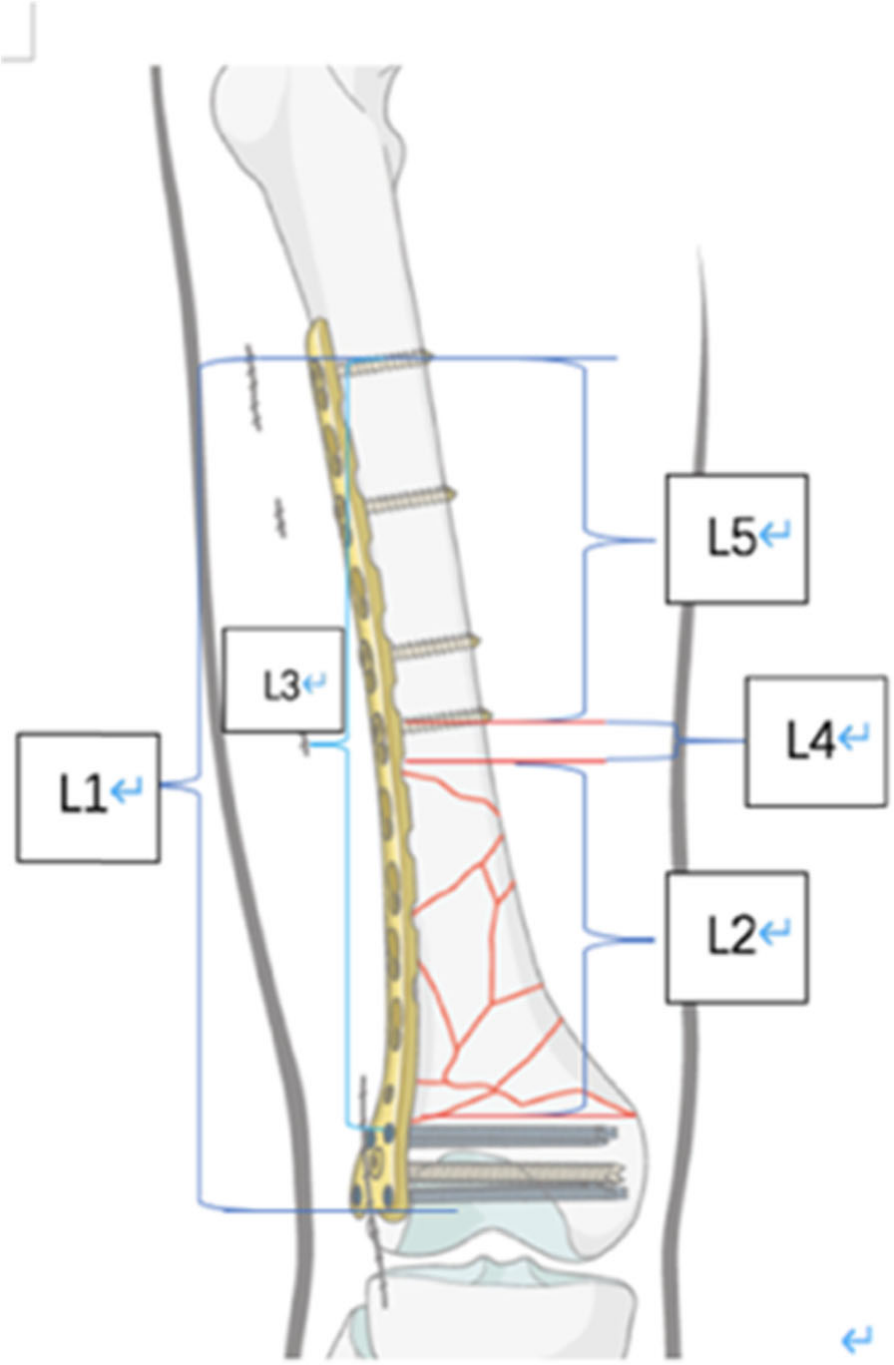
Internal fixation structure. L1, length of plate; L2, length of fracture area; L3, length of plate above condylar screw; L4, distance between proximal part of fracture and screw; L5, working length of proximal plate

The clinical data of evaluation include the following: (1) patient characteristics: gender, age, body mass Index (BMI), comorbidity (diabetes mellitus, steroids use), tobacco, and alcohol addiction; (2) injury-related factors: injury cause, open or closed injury, fracture AO/OTA classification, and supracondylar area involvement; (3) operation-related factors: incision, operation time, and reduction quality; (4) construction of fixation (Fig. 1): length of plate L1/length of fracture area L2 (R1), the length of the plate above the condylar screw L3/the length of the fracture area L2 (R2), density of the proximal condylar screw (D) (the number of screws placed above the proximal condylar screw/the holes), and the distance between proximal part of fracture and screw L4/the working length of the proximal plate L5 (R3).

### Surgical technique and postoperative treatment

All procedures were performed by the senior surgeons and use general or intraspinal anesthesia, supine position, with ilium pillow placed under the hip of the affected side. The lateral approach of para-knee joint or the para-patella was used with a bolster in the supracondylar region. A medial minimally invasive incision was performed to assist reduction according to the reduction. It is important to restore axial alignment, length, and rotation. All patients were fixed with LLP (Synthes, USA). No autogenous iliac bone transplantation was performed in the first operation.

The patients did not need external fixation after operation if the fracture was satisfactorily fixed. Isometric contraction exercise of quadriceps femoris and ankle pump exercise began after recovery of anesthesia. Rehabilitation exercise was carried out after 2 weeks; the injured limb was not loaded within 8 weeks, and the load was gradually increased according to the fracture healing. The X-ray of knee and femur was reexamined at 1, 3, 6, and 12 months after operation and regularly reexamined every 2 months until the fracture healed for delay union patients.

### Statistical analysis

Statistical analysis was conducted with the SPSS 26.0 software (SPSS Inc., USA). Univariate analysis was used to compare the group which received revision and the group that did not. Continuous variables that follow a normal distribution were analyzed using a two-sample Student *t* test; continuous variables that follow a non-normal distribution were analyzed using the Mann Whitney *U* test; Pearson chi-square test and Fisher’s exact test were used to compare the groups with respect to categorical variables. A *P* value < 0.05 was considered to indicate statistical significance.

A logistic regression was applied to identify the significant independent predictors for revision. The full regression model included risk factor candidates based on univariate analysis. Model selection methods such as Wald-backward elimination were used in order to identify important factors from the explanatory variables.

## Results

A total of 152 acute distal femur fracture patients met inclusion criteria. Of these, 16 fractures were surgically revised for nonunion, with a revision rate 10.5%. Patients were divided into non-revision group and revision group. Median follow-up for all 152 fractures was 20 months (range, 9–168 months). Demographic data and the univariate analysis of patient, fracture, first operation, and construct characteristics associated with revision were summarized in Table 1.

**Table 1:** General characteristics and univariate analysis of risk factors for revision after in distal femoral fractures treated with lateral locking plate

Variable	Non revision group	Revision group	*t/z/χ*^2^	*P*
Number	136	16	–	–
Gender (male/female)	28/108	4/12	0.168	0.682*
Age (years)	61.6 ± 14.7	69.0 ± 10.0	− 2.645	0.014^¥^
DM (yes/no)	36/100	6/10	0.871	0.351^£^
Tobacco/alcohol (yes/no)	8/128	2/14	–	0.284*
Steroid usage	6/130	0/16	–	0.507*
BMI	25.4 ± 3.8	27.3 ± 2.1	− 3.005	0.006^¥^
Reason of injury (high/low energy)	80/56	10/6	0.080	0.777^£^
Open/closed	12/124	0/16	–	0.366*
Fracture type (A2/A3/C1/C2/PF)	24/42/10/42/18	0/10/0/2/4	11.223	0.024^£^
Supracondylar involved (no/yes)	70/66	4/12	4.015	0.045^£^
Incision (lateral/lateral + medial)	106/30	8/8	5.961	0.015^£^
Duration of operation (minutes)	144.2 ± 45.9	163.4 ± 55.0	− 1.550	0.123^¥^
Quality of reduction (good/bad)	96/40	5/11	9.937	0.002^£^
R1	3.17 ± 1.43	2.54 ± 0.67	2.997	0.005^¥^
R2	3.31 ± 1.32	2.45 ± 0.72	3.970	0.000^¥^
R3	0.35 ± 0.29	0.19 ± 0.18	2.094	0.038^¥^
Density of supracondylar screws	0.59 ± 0.15	0.65 ± 0.18	− 1.590	0.114^#^

*DM* diabetes mellitus, *BMI* body mass index, *PF* periprosthetic fracture after total knee arthroplasty, *R1* ratio of length of plate/fracture area, *R2* the ratio of the length of the plate/fracture area above the condylar, *R3* ratio of distance between proximal part of fracture and screw/working length of proximal plate

*Fisher’s exact test

^¥^Two-sample Student *t* test

^£^Chi-square test

^#^Mann Whitney *U* test

The values are given as the mean and the standard deviation for continuous variables and as the number of patients for categorical variables

Risk factors for revision in distal femoral fractures treated with LLP were assessed with univariate analysis (Table 1). Significant different factors (*P* < 0.05) were as follows: age, BMI, fracture type, supracondylar involvement, type of incision, quality of reduction, R1, R2, and R3.

Logistic regression was performed in order to simulate a decision analysis. Four out of 9 independent variables were found to have a statistically significant effect on the rate of revision in distal femoral fractures treated with lateral locking plate: age, fracture type, reduction quality, and the ratio of the length of the plate/fracture area above the condylar. Regression coefficients, likelihood ratios, *p* values, adjusted odds ratios, and 95% confidence intervals were determined (Table 2).

**Table 2:** Logistic regression model for predicting revision in patients with distal femoral fractures

Predictors	Regression coefficient	Standard error	Wald *χ*^2^	*P* value	OR	OR 95% CI
Age (X1)	0.096	0.036	7.130	0.008	1.100	1.026–1.180
Type of fracture (X2)			6.750	0.034		
Type of fracture (A3)	2.419	0.854	6.323	0.012	8.572	1.606–45.750
Type of fracture (PF)	2.205	1.024	4.639	0.031	9.073	1.220–67.506
Quality of reduction (X3)	2.036	0.733	7.713	0.005	7.663	1.821–32.253
R2 (X4)	− 1.127	0.425	7.011	0.008	0.324	0.141–0.746
Constant	− 7.879	2.509	9.861	0.002	0.000	–

*PF* periprosthetic fracture after total knee arthroplasty, *R2* ratio of the length of the plate/fracture area above the condylar

The classification of each factor: fracture type X2:1 = A2/C1/C2 fracture, 2 = A3 fracture, 3 = PF; X3:1 = satisfactory reduction, 2 = poor reduction

**Fig. 2:**
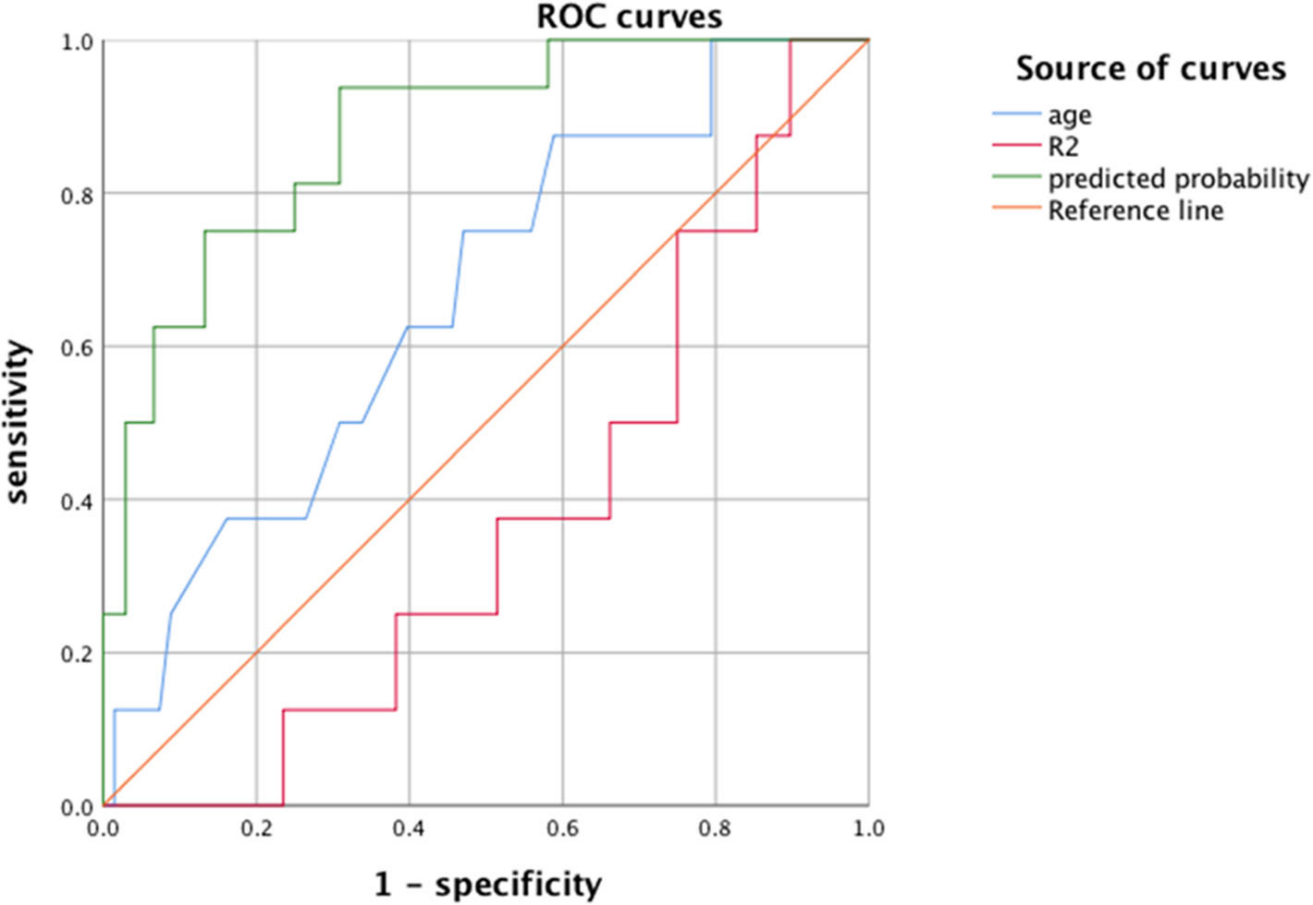
ROC curve analyses were used to evaluate the predictive performance of logistic regression model, age, and R2 to predict revision

**Fig. 3:**
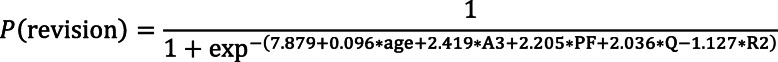
A predictive formula based on the significant risk factors model used to predict the need for revision. PF, periprosthetic fracture after total knee arthroplasty; Q, quality of reduction; R2, ratio of the length of the plate/fracture area above the condylar

A receiver operating characteristic (ROC) curve analysis was used to evaluate the predictive performance of the logistic regression model and its ability to predict the rate of revision after in distal femoral fractures treated with lateral locking plate (Fig. 2). The area under the curve was 0.877, which demonstrated a good diagnostic performance. ROC curve analysis was also used to evaluate age and R2 and its ability to predict the rate of revision (Fig. 2). Age = 61.5, R2 = 1.89, and predicted probability (Fig. 3) *P*(revision) = 0.059 was selected as an optimal cutoff point that best differentiates between patients who should receive revision and those who should not. This cutoff point has the highest sensitivity and specificity rates.

## Discussion

Compared with the traditional angle plate and dynamic condylar plate, the LLP is the most commonly used method nowadays for the treatment of distal femoral fracture for its advantages of minimally invasive, less soft tissue interference, and angular stability [7, 8]. However, there are more and more reports about the complications of LLP in the treatment of distal femoral fracture in recent years [9]. Nonunion of distal femoral fracture is a disastrous complication and seriously affects the quality of life of patients for decreased joint activity and pain. This study attempts to analyze the general situation of patients, fracture-related factors, operation, and construct characteristics to explore the risk factors of revision of LLP in the treatment of distal femoral fracture. The importance of this study is to identify high-risk patients and conduct interventions to promote healing as early as possible, which may reduce the rate of revision in future treatment.

Old age is related to the occurrence and degree of osteoporosis. The probability of internal fixation loosening and fracture nonunion increases in serious osteoporosis patients for the lower holding power of screws [10]. This study suggested similar results; the average age of the non-revision and revision group were 61.6 ± 14.7 years and 69.0 ± 10.0 years respectively; the OR for revision in age > 61.5 group is 4.900 (1.071–22.414).

The principle of “tension band” was used in the treatment of distal femoral fracture with LLP, and the integrity of medial cortex is important and should be restored. AO/OTA type A3 comminuted fracture can cause comminution of medial cortex of metaphysis and destroy its medial supporting ability. In this condition, the tension on the LLP will become repeated bending stress, which can lead to fatigue of the plate; even plate failure or screw loosening, the OR for revision in AO/OTA type A3 group is 8.572 (1.606–45.750), which is consistent with the previous literature [11, 12]. For this reason, some scholars suggest double plate fixation for A3 and C3 type comminuted fractures to improve the fracture healing rate [13].

Periprosthetic fracture after total knee arthroplasty (TKA) is a special type of distal femoral fracture. Although single LLP has less degree of soft tissue damage and certain angle fixation stability, it still may not provide enough stability in this certain condition. In order to overcome these problems, Kim et al. [14] used double plate technique to provide enough stability and reduce the damage of soft tissue as much as possible. This method is especially suitable for distal femoral fracture patients after TKA with poor bone stock, comminuted fracture, and far periprosthetic fracture line [13]. Also, double plated construct had greater stabilization in a simulated fracture model when compared to a single lateral plate [15]. If it is unable to maintain satisfactory alignment and sufficient stability, double plate should be used for fixation in periprosthetic fractures after TKA.

Reduction is the basic AO principle for the treatment of fractures. Poor reduction of distal femoral fractures and residual gap at the fracture end can cause excessive local interfragmentary movement and bone absorption [16]. Also, it cannot restore the support ability of the medial cortex and increase the bending stress of the LLP, which may accelerate fatigue of the plate. Pesciera et al. [17] pointed out that the rate of nonunion could be as high as 12% when the medial alignment was poor and the medial defect was greater than 2 cm. After the concept of biological osteosynthesis (BO) was introduced, protection of soft tissue blood supply at fracture end was widely valued. Therefore, we should restore axial alignment, length, and rotation and try our best to reduce the damage to soft tissue.

Because of the special design of the LLP of the distal femur, its distal shape and the number of screws inserted are constant; therefore, the author thought that the “real” working length of the plate should be considered and introduced the concept of R2: ratio of the length of the plate/fracture area above the condylar. Logistic regression analysis shows that it is a risk factor for revision. Tan et al. [18] pointed out that the insufficient length of the plate may be the risk factor of internal fixation failure for it cannot disperse the stress effectively. The weakest part of LLP is the dynamic hole around the fracture; when the plate concentrates too much stress on a short distance, this part can break out. If there is osteoporosis at the same time, screw loosening and pulling out are more common [19]. In addition, Elkins et al. [20] pointed out that the possible causes of distal femoral fracture nonunion also include too strong LLP structure as to inhibit the movement of fracture end. Therefore, in addition to recent technologies and advances in the management of distal femoral fractures, surgeon needs to always bear in mind the basic principles ruling the plating fixation of distal femur, i.e., apply a sufficiently long plate, maximize the screw fixation to the distal part, and avoid over-rigid fixation [12, 21].

There are still some deficiencies in this study: retrospective study, the sample size is small; there is no analysis of the impact of different surgeon; this paper does not analyze the impact of other potential confounding factors on fracture healing; further prospective randomized controlled clinical trials are needed to verify the results.

## Conclusion

Lateral locking plate is one of the effective internal fixation options in the treatment of distal femoral fracture, but the incidence of complications is not low. Age, fracture type (A3 and periprosthetic fracture after TKA), poor reduction quality, and the ratio of the length of the plate/fracture area above the condylar are the predictive factors for revision after the treatment of distal femoral fracture with LLP. We should choose the appropriate plate and operation strategy according to the type of fracture and patient characteristics to reduce the revision rate. When high-risk cases were identified, interventions should be conducted to promote healing as early as possible and improve the prognosis.

## Data Availability

The datasets used and/or analyzed during the current study are available from the corresponding author on reasonable request.

## Abbreviations

LLP: Lateral locking plate
ROM: Range of motion
EMR: Electronic medical record
BMI: Body mass index
DM: Diabetes mellitus
CI: Confidence intervals
ROC: Receiver operating characteristic
PF: Periprosthetic fracture after total knee arthroplasty
TKA: Total knee arthroplasty
BO: Biological osteosynthesis
